# Cardiac Rehabilitation in German Speaking Countries of Europe—Evidence-Based Guidelines from Germany, Austria and Switzerland LLKardReha-DACH—Part 1

**DOI:** 10.3390/jcm10102192

**Published:** 2021-05-19

**Authors:** Bernhard Rauch, Annett Salzwedel, Birna Bjarnason-Wehrens, Christian Albus, Karin Meng, Jean-Paul Schmid, Werner Benzer, Matthes Hackbusch, Katrin Jensen, Bernhard Schwaab, Johann Altenberger, Nicola Benjamin, Kurt Bestehorn, Christa Bongarth, Gesine Dörr, Sarah Eichler, Hans-Peter Einwang, Johannes Falk, Johannes Glatz, Stephan Gielen, Maurizio Grilli, Ekkehard Grünig, Manju Guha, Matthias Hermann, Eike Hoberg, Stefan Höfer, Harald Kaemmerer, Karl-Heinz Ladwig, Wolfgang Mayer-Berger, Maria-Inti Metzendorf, Roland Nebel, Rhoia Clara Neidenbach, Josef Niebauer, Uwe Nixdorff, Renate Oberhoffer, Rona Reibis, Nils Reiss, Daniel Saure, Axel Schlitt, Heinz Völler, Roland von Känel, Susanne Weinbrenner, Ronja Westphal, on behalf of the Cardiac Rehabilitation Guideline Group

**Affiliations:** 1 Institut für Herzinfarktforschung Ludwigshafen, D-67063 Ludwigshafen, Germany; 2 Zentrum für Ambulante Rehabilitation, ZAR Trier GmbH, D-54292 Trier, Germany; 3 Department of Rehabilitation Medicine, Faculty of Health Sciences Brandenburg, University of Potsdam, D-14469 Potsdam, Germany; annett.salzwedel@fgw-brandenburg.de (A.S.); saraheichler@t-online.de (S.E.); Heinz.Voeller@klinikamsee.com (H.V.); 4 Institut für Kreislaufforschung und Sportmedizin, Abt. Präventive und rehabilitative Sport- und Leistungsmedizin, Deutsche Sporthochschule Köln, D-50937 Köln, Germany; bjarnason@dshs-koeln.de; 5 Department of Psychosomatics and Psychotherapy, Faculty of Medicine, University Hospital, D-50937 Köln, Germany; christian.albus@uk-koeln.de; 6 Institut für Klinische Epidemiologie und Biometrie (IKE-B), Universität Würzburg, D-97078 Würzburg, Germany; k.meng@uni-wuerzburg.de; 7 Klinik Barmelweid AG, CH-5017 Barmelweid, Switzerland; jean-paul.schmid@barmelweid.ch; 8 Reha-Klinik Montafon, A-6780 Schruns, Austria; wbenzer@cable.vol.at; 9 Institute of Medical Biometry and Informatics (IMBI), University of Heidelberg, D-69120 Heidelberg, Germany; matthes.metz@gmail.com (M.H.); Meinhard.kieser@imbi.uni-heidelberg.de (K.J.); dsaure55@hotmail.de (D.S.); 10 Curschmann Klinik Dr. Guth GmbH & Co KG, D-23669 Timmendorfer Strand, Germany; prof.schwaab@drguth.de; 11 Rehabilitationszentrum Großgmain, A-5084 Großgmain, Austria; johann.altenberger@pensionsversicherung.at; 12 Zentrum für Pulmonale Hypertonie, Thorax-Klinik am Universitätsklinikum Heidelberg, D-69126 Heidelberg, Germany; Nicola.Benjamin@med.uni-heidelberg.de (N.B.); ekkehard.gruenig@med.uni-heidelberg.de (E.G.); 13 Institut für Klinische Pharmakologie, Technische Universität Dresden, Fiedlerstraße 42, D-01307 Dresden, Germany; Kurt.Bestehorn@tu-dresden.de; 14 Klinik Höhenried gGmbH, Rehabilitationszentrum am Starnberger See, D-82347 Bernried, Germany; christa.bongarth@hoehenried.de (C.B.); hans-peter.einwang@hoehenried.de (H.-P.E.); 15 Alexianer St. Josefs-Krankenhaus Potsdam-Sanssouci, D-14471 Potsdam, Germany; g.doerr@alexianer.de; 16 Deutsche Rentenversicherung Bund (DRV-Bund), D-10709 Berlin, Germany; dr.johannes.falk@drv-bund.de (J.F.); susanne.weinbrenner@drv-bund.de (S.W.); 17 Reha-Zentrum Seehof der Deutschen Rentenversicherung Bund, D-14513 Teltow, Germany; reha-klinik.seehof@drv-bund.de; 18 Klinikum Lippe, Standort Detmold, D-32756 Detmold, Germany; stephan.gielen@klinikum-lippe.de; 19 Universitätsbibliothek, Universitätsmedizin Mannheim, D-68167 Mannheim, Germany; grillimaurizio@posteo.de; 20 Reha-Zentrum am Sendesaal, D-28329 Bremen, Germany; dr.guha@rehaklinik-sendesaal.de; 21 Klinik für Kardiologie, Universitätsspital Zürich, Rämistrasse 100, CH-8091 Zürich, Switzerland; matthias.hermann@usz.ch; 22 Wismarstraße 13, D-24226 Heikendorf, Germany; eike.hoberg@t-online.de; 23 Universitätsklinik für Medizinische Psychologie und Psychotherapie, Medizinische Universität Innsbruck, A-6020 Innsbruck, Austria; stefan.hoefer@i-med.ac.at; 24 Klinik für Angeborene Herzfehler und Kinderkardiologie, Deutsches Herzzentrum München, Klinik der Technischen Universität München, D-80636 München, Germany; Kaemmerer@dhm.mhn.de; 25 Department of Psychosomatic Medicine and Psychotherapy, Klinikum rechts der Isar, Technische Universität München (TUM) Langerstraße 3, D-81675 Munich, Germany; karl-heinz.ladwig@tum.de; 26 Klinik Roderbirken der Deutschen Rentenversicherung Rheinland, D-42799 Leichlingen, Germany; wolfgang.mayer-berger@klinik-roderbirken.de; 27 Cochrane Metabolic and Endocrine Disorders Group, Institute of General Practice (ifam), Medical Faculty of the Heinrich-Heine University, Werdener Straße. 4, D-40227 Düsseldorf, Germany; maria-inti.metzendorf@med.uni-duesselorf.de; 28 Hermann-Albrecht-Klinik METTNAU, Medizinische Reha-Einrichtungen der Stadt Radolfzell, D-73851 Radolfzell, Germany; Roland.Nebel@mettnau.com; 29 Institut für Sportwissenschaft, Universität Wien, Auf der Schmelz 6 (USZ I), AU-1150 Wien, Austria; rhoia.neidenbach@univie.ac.at; 30 Universitätsinstitut für Präventive und Rehabilitative Sportmedizin, Uniklinikum Salzburg Paracelsus Medizinische Privatuniversität, A-5020 Salzburg, Austria; j.niebauer@salk.at; 31 EPC GmbH, European Prevention Center, Medical Center Düsseldorf, D-40235 Düsseldorf, Germany; nixdorff@epccheckup.de; 32 Lehrstuhl für Präventive Pädiatrie, Fakultät für Sport- und Gesundheitswissenschaften, Technische Universität München, D-80992 München, Germany; renate.oberhoffer@tum.de; 33 Kardiologische Gemeinschaftspraxis Am Park Sanssouci, D-14471 Potsdam, Germany; rona.reibis@hotmail.de; 34 Schüchtermann-Schiller’sche Kliniken, Ulmenallee 5-12, D-49214 Bad Rothenfelde, Germany; nreiss@schuechtermann-klinik.de; 35 Paracelsus Harz-Klinik Bad Suderode GmbH, D-06485 Quedlinburg, Germany; axel.schlitt@pkd.de; 36 Klinik am See, D-15562 Rüdersdorf, Germany; 37 Klinik für Konsiliarpsychiatrie und Psychosomatik, Universitätsspital Zürich, CH-8091 Zürich, Switzerland; roland.vonkaenel@usz.ch; 38 Herzzentrum Segeberger Kliniken, D-23795 Bad Segeberg, Germany; ronja.westphal@segebergerkliniken.de

**Keywords:** cardiac rehabilitation standards, scientific guidelines, secondary prevention, coronary artery disease, chronic heart failure, heart valve repair, ICD-CRT, ventricular assist device, heart transplantation, peripheral artery disease, pulmonary hypertension, myocarditis, adults with congenital heart disease

## Abstract

Background: Although cardiovascular rehabilitation (CR) is well accepted in general, CR-attendance and delivery still considerably vary between the European countries. Moreover, clinical and prognostic effects of CR are not well established for a variety of cardiovascular diseases. Methods: The guidelines address all aspects of CR including indications, contents and delivery. By processing the guidelines, every step was externally supervised and moderated by independent members of the “*Association of the Scientific Medical Societies in Germany”* (AWMF). Four meta-analyses were performed to evaluate the prognostic effect of CR after acute coronary syndrome (ACS), after coronary bypass grafting (CABG), in patients with severe chronic systolic heart failure (HFrEF), and to define the effect of psychological interventions during CR. All other indications for CR-delivery were based on a predefined semi-structured literature search and recommendations were established by a formal consenting process including all medical societies involved in guideline generation. Results: Multidisciplinary CR is associated with a significant reduction in all-cause mortality in patients after ACS and after CABG, whereas HFrEF-patients (left ventricular ejection fraction <40%) especially benefit in terms of exercise capacity and health-related quality of life. Patients with other cardiovascular diseases also benefit from CR-participation, but the scientific evidence is less clear. There is increasing evidence that the beneficial effect of CR strongly depends on “treatment intensity” including medical supervision, treatment of cardiovascular risk factors, information and education, and a minimum of individually adapted exercise volume. Additional psychologic interventions should be performed on the basis of individual needs. Conclusions: These guidelines reinforce the substantial benefit of CR in specific clinical indications, but also describe remaining deficits in CR-delivery in clinical practice as well as in CR-science with respect to methodology and presentation.

## 1. Introduction

Cardiovascular diseases (CVD) represent the leading cause of premature death and disability in Europe and other countries all over the world. Moreover, the socioeconomic burden of CVD is significant [1,2]. It is well accepted that primary and secondary prevention represent a therapeutic key approach to reduce incidence and prevalence of morbidity and mortality from CVD [3,4]. Cardiovascular rehabilitation (CR) represents a multidisciplinary therapeutic intervention to accomplish these objectives, but in clinical practice CR-delivery still is not sufficiently established neither in Europe nor in other countries of the world [5,6]. Although CR-quality has been graded to be high in a recent global survey, this adjudication needs to be questioned, as content, volume and intensity of CR-delivery are not binding in many countries and substantially vary even in between industrial, high income countries [5,7,8,9]. Until now, these deficits have not satisfactorily been solved, despite the availability of several scientific guidelines and expert position papers [5,10,11,12,13,14,15].

Within the past years, however, an increasingly targeted approach in rehabilitation science was achieved to more precisely define CR-strategies and components, which aim to successfully improve long-term clinical outcomes especially in patients with coronary artery disease (CAD) [7,16,17,18,19,20,21,22,23]. Based on these data it is possible to more exactly define minimal requirements for successful CR-delivery not only in terms of reducing morbidity and mortality but also in terms of improving quality of life and ensuring an active and long-term social (re)integration. Importantly, this scientific progress needs to be expanded by including all clinically relevant cardiovascular diseases potentially being qualified for CR-participation, as most of them imply chronic diseases to be stabilized and attenuated.

Against this background the aims of the newly developed Cardiac Rehabilitation Guidelines of German Speaking Countries in Europe (LLKardReha-DACH) can be summarized as follows:

- To newly define the clinical indications for CR-referral, and to describe actual scientific evidence and minimal requirements to be successful in improving CR-outcomes in terms of clinical prognosis, quality of life and other health-related qualities.
- To evaluate a broad spectrum of cardiovascular diseases and discuss all aspects of CR delivery including an individualized therapeutic approach, psychosocial aspects, individual education and long-term adherence to prevention of CV diseases.
- To harmonize CR within the area of Germany, Austria and Switzerland by CR-content and delivery in detail as adapted to the individual’s diseases and needs.
- To strictly follow a scientific approach with predefined procedures in a literature search, evidence generation, data presentation and consenting recommendations, all steps being externally supervised by the *“Association of the Scientific Medical Societies in Germany”* (AWMF) as an independent institution.
- To clearly define progress and actual deficits in CR-delivery and scientific approaches.

The recommendations of the guidelines are presented and discussed in two parts. The first part, presented in this publication, describes the methods in evidence generation and consenting recommendations. This is followed by the presentation and discussion of a broad range of potential indications for CR-referral and delivery. The second part, being published separately, concentrates on CR-contents including medical supervision, individually adapted physical exercise, secondary prevention implementation, psychosocial aspects and special forms of CR. The extended guidelines in the German language are published by the AWMF, https://www.awmf.org/leitlinien/aktuelle-leitlinien.html (17 January 2021).

## 2. Methods

### 2.1. Guidelines Initiation, Organization, Financial Support and Scientific Supervision

The guidelines were initiated, sponsored and organized by the *“German Society for Cardiovascular Prevention and Rehabilitation”* (DGPR), whereby all organizational steps were performed in close cooperation with the “*Austrian Society of Cardiology*” (“*Working Group for Prevention, Rehabilitation and Sports Cardiology*”), and the “*Swiss Working Group for Cardiovascular Prevention, Rehabilitation and Sports Cardiology*” (SCPRS).

The lead management took responsibility for the guideline development, contents, presentation and outlay. The lead management also initiated and supervised the selection of authors with topic-related expertise and was responsible for the internal control and pre-reviewing of all contents (Table 1).

The steering committee responsible for the scientific supervision of guideline generation and contents, finally consented to the evidence-based recommendations. The steering committee consisted of representatives of the most relevant medical societies representing cardiovascular diseases in Germany, Austria and Switzerland, and additionally of a representative of the German pension funds (Table 1).

Patient’s representatives (*n* = 6), selected on a voluntary basis and supported by the “German Heart Foundation”, were also included in guideline processing for implementation of “shared decision making”.

The external scientific supervision of guideline generation, formulation and grading of evidence-based recommendations was delivered by the AWMF (Table 1).

The constitutive meeting of the steering committee was held on 24 September 2014. The guideline was finalized in German language and published online in January 2020 (www.awmf.org, accessed on 17 January 2021)

### 2.2. Guidelines Generation and Reviewing

All participating authors worked on a voluntary basis and declared their written conflicts of interest. The guideline strictly followed the rules and regulations of the AWMF, who also supervised guideline generation and methodological aspects. The scientific grading process also was predefined and moderated by specialists of the AWMF.

The meta-analyses associated with these guidelines and all biometrical aspects were supported by the Institute of Medical Biometry and Informatics at the University of Heidelberg (IMBI).

By decision of the steering committee, the generation of scientific evidence followed three approaches (for details see Table 2):

- Topic-related, a structured literature search and review followed by a meta-analysis;
  (classification “S3” = “high grade evidence”)
- Topic-related semi-structured literature search and review without meta-analysis, recommendations by a predefined and supervised consenting process;
  (classification “S2k” = medium grade evidence)
- Topic-related summaries of the most recent scientific guidelines published by national and international medical societies (NER = narrative evidence reporting).

### 2.3. Contents, Presentation of Evidence-Based Clinical Recommendations and Classification of Evidence

The content of the guideline was defined by the steering committee and included the objectives and tasks of the guidelines, the methodology being applied for evidence generation, legal regulations and healthcare structures determining rehabilitation delivery in Germany, Austria and Switzerland. This was followed by the presentation of the primary objectives and tasks of cardiac rehabilitation including individual goal setting, cardiovascular prevention, and psychosocial interventions. The main chapters outlined CR-indications, CR-contents including a detailed presentation of exercise training modalities and education, special aspects of gender, age and minorities, long-term prevention services, and special prevention and rehabilitation concepts (see Table S1, Supplemental Material).

The “grades of recommendation” were based on the scientific evidence (for the methods of evidence generation see Table 2) and a formal consensus process of all members of the steering committee supervised by the AWMF.

In accordance with the “GRADE Evidence-to-Decision framework” grading of recommendations was based on the following criteria [31]

- Relevance of outcomes and quality of evidence for each relevant outcome;
- Consistency of study results;
- Directness/applicability of the evidence to the target population, PICO specifics (Population(s), Intervention(s), Control(s), Outcome(s));
- Precision of effect estimates reg. confidence intervals;
- Magnitude of the effects;
- Balance of benefit and harm;
- Ethical, legal, economic considerations;
- Patient’s preferences.

The “grades of recommendation” are outlined in Table 3. The “degree of consensus” is expressed in percentages of all steering committee members participating in the consensus generating process.

The “classification of scientific evidence” followed the definitions of SIGN (Scottish Intercollegiate Guidelines Network [30] and only was applicated in chapters classified by “S3” (see Table 2).

Within this system the number “1” refers to randomized controlled studies (RCTs) or meta-analyses of RCTs, number “2” refers to controlled cohort studies or meta-analyses with controlled cohort studies, “3” reflects non-analytical studies and “4” refers to “expert opinions”.

Additionally, the risk of bias is graded as being very low (++), low to moderate (+) or high (−). For a detailed description of the SIGN classification of scientific evidence see the supplemental materials.

## 3. Results and Evidence-Based Recommendations for CR-Initiation and Delivery

The available data from various sources to mirror CR-delivery in clinical practice in German speaking countries are incomplete. Still, these data allow the following conclusions: Patients with coronary artery disease represent the vast majority of patients participating in cardiovascular rehabilitation followed by patients after heart valve repair. In contrast, patients with chronic heart failure, diseases of the aorta, peripheral artery disease and other potential indications only represent a minority [32,33].

In the following, the recommendations for cardiovascular rehabilitation initiation and delivery are outlined and discussed. The diseases being evaluated can be categorized as follows:

- Atherosclerotic cardiovascular diseases;
- Heart failure;
- Diseases of the aorta;
- Diseases of the pulmonary vessels;
- Myocarditis;
- Congenital heart diseases.

### 3.1. Patients after Acute Coronary Syndrome (ACS)

The following recommendations are based on the newly performed meta-analysis Cardiac Rehabilitation Outcome Study (CROS)-I and CROS-II in addition to other recently published meta-analyses and trials (classification: **S3**, see Section 2, “Methods”).

#### 3.1.1. Recommendations

- After acute coronary syndrome (STEMI, NSTEMI or unstable angina pectoris) patients are recommended to participate in cardiac rehabilitation (CR). This is based on the scientific evidence of CR-participation being associated with a significant reduction in total mortality, cardiac mortality and re-infarction rate (↑↑, 1++, 100%) [7,16,17,18,27];
- After ACS cardiac rehabilitation is recommended to start as early as possible, but not later than 3 months after hospital discharge, to be center-based (ambulatory, residential or mixed) and to be under supervision and responsibility of expert cardiologists. (↑↑, 2++, 100%) [7,17,27]. “Centre-based CR” is defined as cardiovascular rehabilitation under the supervision and responsibility of a rehabilitation center.
- CR is recommended to be based on a structured, supervised and individually adapted exercise program to sustainably increase the individual’s exercise capacity (↑↑, 1++, 100%) [17,27,34,35,36,37,38].
- To be successful, cardiac rehabilitation of patients after ACS is recommended to meet the following minimal requirements (↑↑, 1++, 100%) [7,21,38,39]:
  - Total exercise volume to be ≥1000 min as calculated by the “number of weeks” times “exercise sessions per week” times “exercise duration per session in minutes”;
  - Exercise intensity to be within the upper third of the individually achieved and clinically tolerable range (as measured in METs; VO_2max_; Watt; RPE)
  - The number of rehabilitation sessions including exercise, information, education and psychosocial interventions to be ≥36.
- In addition to exercise training programs, CR is recommended to provide the following components being individualized to the patient’s needs and preferences (↑↑, 2++, 100%) [16,17,18,27]: consequent management of cardiovascular risk factors and risk diseases including pharmacotherapy, information, motivation, education, psychological support and intervention, as well as support to regain and/or keep social and vocational integration.

#### 3.1.2. Scientific Evidence

The beneficial prognostic effect of CR after ACS primarily is based on the Cardiac Rehabilitation Outcome Study (CROS). CROS evaluated the prognostic effect (total mortality as primary endpoint) of center-based and multicomponent CR after ACS (50,653 patients out of 15 studies published 1995 or later) [17,27].

The results of CROS are in line with other recent meta-analyses [7,16,18,21]. Although these analyses varied in their inclusion criteria, especially with respect to CR-definitions and contents, all focused on minimal requirements for CR-delivery to be successful in improving prognosis [7,16,17,18,21,27].

In addition, the results of numerous randomized controlled studies (RCT) clearly show the beneficial effect of individually adapted exercise training in improving prognosis, provided the individually adapted exercise volume does not undercut a minimum as defined in the recommendations above [34,35,37,38].

The effect of CR-participation on the health-related quality of life has been evaluated in several studies, being summarized by a Cochrane analysis in 2016. The methodology applied showed a considerable heterogeneity, hence a meta-analysis has not been performed. Still, a trend of CR-participation being associated with an improved health-related quality of life was shown [7].

#### 3.1.3. Limitations

CROS is primarily based on controlled cohort studies. Only one RCT (RAMIT including 1813 patients after ACS randomized between “CR-delivery” and “usual care”) fulfilled the CROS-inclusion criteria, but showed neutral results with respect to mortality during a follow-up of two years [40]. However, as the RAMIT sample size only represents about 23% of the anticipated sample in each trial arm, a significant under-powering may limit the validity of this trial [17,40].

Rehabilitation delivery and content considerably vary between the studies under evaluation with respect to supervision, exercise volume and individual support, and a detailed reporting of CR-contents in some studies is limited [17,27].

With respect to the CR-effect on “social participation”, data are rare. The individual risk of “vocational reintegration failure” should be identified during CR-participation as individually adapted and work-related interventions may support vocational reintegration [41,42].

With respect to the effect of CR-participation after ACS on “cost-effectiveness” data are rare and partly inconsistent. Moreover, study designs were considerably heterogeneous, thereby not allowing solid conclusions [7,43,44].

### 3.2. Patients after Coronary Bypass Surgery (CABG)

The following recommendations are based on the newly performed meta-analysis CROS-I and CROS-II in addition to other recently published meta-analyses and trials (classification: **S3**, see Methods).

#### 3.2.1. Recommendations

- After coronary bypass surgery (CABG) patients are recommended to participate in cardiac rehabilitation (CR) to improve the patient’s prognosis by reducing total mortality (↑↑, 2++, 100%) [17,27].
- After CABG cardiac rehabilitation is recommended to start as early as possible, but not later than 3 months after hospital discharge, to be center-based (ambulatory, residential or mixed) and to be under supervision and responsibility of expert cardiologists (↑↑, 2++, 100%) [17,27].
- CR is recommended to be based on a structured, supervised and individually adapted exercise program to sustainably increase the individual’s exercise capacity
- (↑↑, 1++, 100%) [17,27,34,35,37,38].
- To be successful, cardiac rehabilitation after CABG is recommended to meet the following minimal requirements (↑↑, 2++, 100%) [7,21,38,39,45,46,47]:
  - Control of post-surgery risks like sternum instability, delayed wound healing, pneumonia or post-thoracotomy syndrome—if needed immediate treatment in cooperation with the heart center being responsible;
  - Total exercise volume to be ≥1.000 min as calculated by the “number of weeks” times “exercise sessions per week” times “exercise duration per session in minutes”;
  - Exercise intensity to be within the upper third of the individually achieved and clinically tolerable range (as measured in METs; VO_2max_; Watt; PRE);
  - The number of rehabilitation sessions including exercise, information, education and psychosocial interventions to be ≥36.
- In addition to exercise training programs, CR is recommended to provide the following components individualized to the patient’s needs and preferences (↑↑, 2++, 100%):
  - Consequent management of cardiovascular risk factors and diseases including pharmacotherapy;
  - Information, motivation, education, psychological support and intervention;
  - Support to regain and/or keep social (re)integration and vocational reintegration [18,45,46,47].

#### 3.2.2. Scientific Evidence

The beneficial prognostic effect of CR after CABG primarily is based on the Cardiac Rehabilitation Outcome Study (CROS). CROS evaluated the prognostic effect (total mortality as primary endpoint) of center-based and multicomponent CR after CABG (14,583 patients out of six studies published in 1995 or later) [17,27].

The minimal requirements for CR to be successful in patients after CABG are based on the inclusion criteria defined by CROS, on the need to consequently treat/eliminate cardiovascular risk factors, and to deliver an individually adapted minimal volume of a structured and supervised exercise training [7,17,18,21,27,38]. The recommendations considering the special requirements of post-surgery patients are in line with recently published international guidelines [45,46,47]

Apart from the meta-analyses evaluating exercise-based and multicomponent CR, numerous controlled studies consistently show the beneficial effect of individually adapted exercise training in improving prognosis, provided that the individually adapted exercise volume does not undercut a minimum as defined in the recommendations above [33,34,36,37,38].

The effect of CR-participation on the health-related quality of life has been evaluated in several studies being summarized by a Cochrane analysis in 2016. The methodology applied showed a considerable heterogeneity, hence a meta-analysis has not been performed. Still, a trend of CR-participation being associated with an improved quality of life was shown [7].

Factors potentially obstructing vocational reintegration (e.g., prolonged hospitalization, persistent angina pectoris, dissatisfactory vocational situation, depression) clearly can be identified during CR [47]. CR-programs simulating job situation may support vocational reintegration [41]. In a study performed in Israel (*n* = 2085 patients after CABG) only 6.9% participated in CR. However, vocational reintegration of CR-participants significantly exceeded that of the controls [48].

#### 3.2.3. Limitations

The number of controlled studies specifically investigating the prognostic effect of CR following CABG is small. Although patients after CABG represent a special entity including specific risks, “mixed CAD study populations” are used in many studies thereby potentially concealing clinical and prognostic effects of cardiovascular rehabilitation in well-defined and clinically important subgroups [27,49].

The studies evaluating the prognostic effect of CR in patients after CABG are considerably heterogeneous with respect to content, duration and intensity, thereby potentially hampering interpretation of the results.

With respect to the CR-effect on social and vocational reintegration, there are no studies in German speaking countries in Europe. International available studies show effects, but the results cannot be transferred due to different social systems and living conditions [41,48,50].

### 3.3. Patients with Chronic Coronary Syndrome (CCS)

The following recommendations are based on the information delivered by newly performed semi-structured literature searches (classification: S2k, see Methods).

#### 3.3.1. Recommendations

- Patients with CCS with or without PCI in history are recommended to participate in cardiac rehabilitation (CR), provided that one or more of the following preconditions are fulfilled (↑↑, 88%):
  - The clinical prognosis is limited due to insufficiently treated cardiovascular risk factors and risk diseases (e.g., ESC-SCORE > 5%);
  - Ongoing typical cardiac symptomatology (angina pectoris, dyspnoe) but no option for (additional) coronary revascularizations;
  - Comorbidities including PAD, COPD, diabetes, chronic renal disease increasing the risk of adverse outcomes;
  - Increased risk of CCS to jeopardize the individual’s social and vocational reintegration during follow-up.
- To be successful, CR in patients with CCS is recommended to meet the following structural baseline conditions (↑↑, 100%):
  - To be center-based (out-patient, in-patients, mixed) and under the supervision and responsibility of a cardiologist;
  - To be based on a structured, supervised and individually adapted exercise program in order to sustainably increase the individual’s physical performance.
- In addition, the CR-program is recommended to meet the following minimal requirements of CR-implementation (↑↑, 100%):
  - Total exercise volume to be ≥1000 min as calculated by the “number of weeks” times “exercise sessions per week” times “exercise duration per session in minutes”;
  - Exercise intensity to be within the upper third of the individually achieved and clinically tolerable range (as measured in METs; VO_2max;_ Watt);
  - The number of rehabilitation sessions including exercise, information, education and psychosocial interventions to be ≥36.
- In addition to exercise training, the CR-program is recommended to provide the following components to be individually adapted to the needs of the patients with CCS (↑↑, 100%):
  - Information, motivation, education, psychological support, and support in social and vocational reintegration;
  - Strict treatment/reduction of cardiovascular risk factors and risk diseases.

#### 3.3.2. Scientific Evidence

The prognostic effect of CR-participation in patients with chronic coronary syndromes is less well documented. The Cardiac Rehabilitation Outcome Study (CROS) especially evaluated patients after ACS, after CABG and a mixed CAD-population, but CCS-patients as a single population were not examined [27]. The same is true with regard to the latest Cochrane analysis evaluating the prognostic effect of CR [7]. In another small Cochrane review including 581 patients (seven RCTs, quality being graded as “low”) with exclusively stable angina, a significant increase in exercise capacity of CR-participants could be shown, but neither “total mortality” nor “re-infarction” or “re-hospitalization rates” were reduced [51].

Several RCTs of various sizes evaluated the clinical effects of a variety of “preventions programs” not comparable to cardiovascular rehabilitation as defined in this guideline. Nevertheless, these studies showed improvements in “health-related quality of life”, “exercise capacity”, and “adherence to secondary prevention measures” (e.g., exercise, diet, smoking cessation, medication, psychological parameters, etc.), but no significant improvements in clinical prognosis have been shown [52,53,54,55,56,57,58,59,60,61]. In addition, some RCTs evaluated the effect of variable-defined rehabilitation/prevention programs after elective PCI in patients with CCS showing improved lifestyle measures and a significantly clinical prognosis (CV-mortality and combined endpoint of CV-mortality, ACS, CABG, PCI) [62,63].

#### 3.3.3. Limitations

CR-programs applied to evaluate the effect of CR in CCS patients show a considerable variability in contents and delivery and do not always match the strict requirements as outlined in this guideline. Moreover, data on the prognostic effects of CR-programs in these CCS patients are limited; therefore, a critical selection of CCS-patients for CR-referral as well as the application of a strictly supervised and multicomponent CR-program addressing the individual patient’s needs is warranted.

### 3.4. Patients with High or Very High Cardiovascular Risk but without Witnessed Coronary Artery Disease

The following recommendations are based on the information delivered by newly performed semi-structured literature searches (classification: S2k, see Methods).

#### 3.4.1. Recommendations

- All patients with high or very high cardiovascular risk (CVR, ESC-SCORE ≥ 5) but without witnessed CAD are recommended to participate in structured and multicomponent prevention programs (e.g., provided by employers, health agencies, pension funds and others) (↑↑, 100%).
- In patients with high or very high cardiovascular risk (CVR, ESC-SCORE ≥ 5) but without witnessed CAD, participation in a multidisciplinary CR-program is suggested, if the following preconditions are fulfilled (↑, 100%):
  - Presence of modifiable cardiovascular risk factors;
  - High motivation of the individual to reduce CV-risk and improve healthy lifestyle;
  - The individual’s social and vocational integration/reintegration being at risk.

#### 3.4.2. Scientific Evidence

There is sufficient evidence that participation of individuals with high cardiovascular risk in multicomponent and long-term prevention programs is associated with a risk reduction and a reduction in adverse CV-events. Importantly, the individuals need to be actively integrated into these programs (“shared decision”), and potential psychosocial issues also need to be addressed [4,64,65,66,67,68,69].

#### 3.4.3. Limitations

There are no scientific data on the prognostic effect of primary prevention as delivered by short term CR in Germany. To assure long-term effects in risk reduction patients need to be encouraged to also participate in subsequent long-term programs.

### 3.5. Patients with Chronic Heart Failure

The following recommendations are based on the newly performed meta-analysis CROS-HF in addition to other recently published meta-analyses and trials (classification: **S3**, see Methods).

The term “chronic heart failure” reflects a heterogeneous disease entity. The following recommendations primarily refer to patients with systolic myocardial failure (heart failure with reduced ejection fraction, HFrEF) of various origins being clinically stabilized (NYHA I–III).

#### 3.5.1. Recommendations

- Patients with chronic heart failure (NYHA I-III) and clinically stabilized patients after acute decompensated heart failure are recommended to participate in CR, as CR attendance is associated with an improvement of exercise capacity, functional capacity and improved health-related quality of life (↑↑, 1+, 100%) [4,28,70,71,72,73,74,75].
- For patients with chronic heart failure (CHI, NYHA I-III) and for stabilized patients after acute decompensated heart failure, cardiac rehabilitation is recommended to start as early as possible after hospital discharge (↑↑, 1+, 100%) [14,28,70,72,74].
- CR is recommended to be center-based (out-patient, in-patient or mixed types), multidisciplinary and to be under supervision and responsibility of expert cardiologists (↑↑, 1+, 100%) [14,28,70,72,73,74].
- CR-delivery is recommended to be based on an individually adapted, structured and supervised exercise training program (↑↑, 1+, 100%) [72,73,75].
- In addition to individualized and supervised exercise training, CR in chronic heart failure patients is recommended to include the following components:
  - Strict management of cardiovascular risk factors and risk diseases including a sustainable implementation of guideline-adjusted medication (↑↑, 1++, 100%) [72,74];
  - Information and education to improve patient’s comprehension and self-management (↑↑, 1+, 100%);
  - Psychological support and if needed psychological interventions in order to improve coping, self-efficacy and to overcome depression and anxiety (↑↑, 4 “expert opinion”, 100%).

#### 3.5.2. Scientific Evidence

There is well documented evidence that participation of clinically stabilized patients with heart failure NYHA I-III in a medically supervised, center-based, multicomponent CR is associated with a significant improvement of exercise and functional capacity associated with an increase in health-related quality of life [28,71,72,73,74,75]. In patients with HFrEF and a LV-EF ≤ 40% there is no evidence that participation in a multicomponent CR-program is associated with either a significantly reduced total mortality or a reduced cardiac mortality [28,75]. During a follow-up of more than 12 months, CR-participation also does not have an effect on the rates of rehospitalization in this population [28].

#### 3.5.3. Limitations

A clinically beneficial effect of CR-participation of severely diseased HFrEF patients strongly depends on the close supervision and monitoring of these patients. The failure of LV-EF improvement despite a significant increase in exercise capacity and quality of life is not yet fully explained and further investigations are warranted [28].

Causes of rehospitalization of CHF-patients not necessarily reflect worsening of heart failure, and therefore may be an endpoint that is difficult to interpret [28].

With respect to CR-delivery in CHF-patients in clinical practice, there is an urgent need to define international standards. This also is true for scientific standards to evaluate specific therapeutic interventions during CR-participation.

### 3.6. Patients after Surgical or Interventional Heart Valve Repair

The following recommendations are based on the information delivered by newly performed semi-structured literature searches (classification: S2k-, see Section 2).

#### 3.6.1. Recommendations

- Patients after surgical heart valve replacement or heart valve repair as well as patients after interventional heart valve replacement are recommended to participate in CR (↑↑, 100%).
- In all patients after heart valve repair CR is recommended to provide a detailed non-invasive cardiovascular check-up including a stress test and echocardiogram at the beginning of CR. In case of clinical signs of endocarditis, a transoesophageal echocardiogram is recommended to be performed immediately for further evaluation (↑↑, 100%).
- In frail and older patients after heart valve repair mobility and functional capacity are suggested to be checked (6-min walking test; “Timed-Up & Go” test; “hand-grip strength”) in addition, which in some patients may be supplemented by the evaluation of cognitive performance (e.g., Mini Mental State Examination), (mal)nutrition (“Mini Nutritional Assessment”), and activities of daily life (e.g., Barthel Index) (↑, 100%).
- Moreover, all patients after heart valve repair are recommended to be evaluated with respect to their “health-related quality of life”, potential “risk behaviors” and “psychosocial issues” as a basis for individualized therapeutic, psychological and/or vocational support during CR (↑↑, 100%).
- Exercise training of patients after heart valve repair is recommended and should include an individually adapted combination of structured endurance training, dynamic resistance training and special training to improve coordination and flexibility as well as coordination, especially in older patients (↑↑, 100%).
- All patients after heart valve repair are recommended to be informed on prophylaxis of endocarditis, and—if appropriate—on the consequences of anticoagulation and thoracotomy (↑↑, 100%).
- Patients, who need to be anticoagulated with vitamin K antagonists, are suggested to participate in a training course of INR self-monitoring (↑, 100%).

#### 3.6.2. Scientific Evidence

Based on observational and randomized controlled studies as well as on data delivered by two systematic reviews and meta-analyses, CR-participation after surgical or interventional heart valve repair is associated with a significant increase in the individual’s exercise capacity, health related quality of life and individual autonomy, whereas anxiety is reduced [76,77,78,79,80,81,82,83].

#### 3.6.3. Limitations

There are no controlled studies investigating the prognostic effect of cardiovascular rehabilitation following heart valve repair. Moreover, a considerable need remains to further investigate and to further specify CR-content and delivery (e.g., intensity of medical supervision needed, individualization of therapeutic measures, implementation of modern communication techniques like tele-medicine).

### 3.7. Patients after Implantation of a Cardioverter-Defibrillator (ICD), Resynchronisation System (CRT), and Patients with a Wearable Cardioverter-Defibrillator (WCD)

The following recommendations are based on the information delivered by newly performed semi-structured literature searches (classification: S2k, see Methods).

#### 3.7.1. Recommendations

- Patients after ICD, CRT, or WCD implantation are recommended to participate in cardiac rehabilitation (CR) (↑↑, 100%).
- CR is recommended to include individually adapted endurance and strength training of low to moderate intensity depending on the nature of the underlying cardiac disease and the actual clinical status (↑↑, 100%).
- In patients with recently implanted devices (<6 weeks) training intensity is suggested to be extremely light (RPE 6–8/20) (↑, 100%). In case of an ongoing clinically stable follow-up beyond 6 weeks after device implantation, exercise intensity is recommended to be stepwise and individually adapted depending on the disease related functional limitations as evaluated by echocardiography, stress test, and Holter-ECG (↑↑, 100%).
- Within the first 6 weeks after device implantation, the ipsilateral pectoral girdle is recommended to be spared in order to avoid electrode dislocation (↑↑, 100%).
- Moreover, it is suggested to avoid inappropriate strain on this particular region also in the long run (↑, 100%).
- ICD-patients and all therapeutic personnel being in contact with them are recommended to be aware of the individually programmed “anti-tachycardia pacing” (ATP) heart rate (“ICD detection rate”). To avoid inadequate ICD shock delivery the maximum heart rate must be kept at least 10 -20 beats per minute below the ICD-ATP rate (↑↑, 100%).
- Direct exposures of the ICD-CRT-pulse generator to mechanical stress are recommended to strictly be avoided (↑↑, 100%).
- In WCD-patients exercise training also is suggested to be adapted to the underlying heart disease and follow the recommendations concerning ICD-patients (↑, 100%).
- There are several other specific features of ICD/CRT/WCD patients like anxiety and/or depression and post-traumatic stress disorder that are recommended to be considered, and patients and their dependents need to be informed about this (↑↑, 100%).

#### 3.7.2. Scientific Evidence

Taking into account the individual exercise capacity, participation of ICD/CRT-patients in cardiac rehabilitation has been shown to be safe in general [84,85,86,87,88,89]. Notably, in patients with sedentary behavior there may be some increased risk of ventricular arrhythmia within the first hour of exertion [90]. However, regular aerobic exercise training for 8 weeks was associated with a significant increase in exercise capacity. Moreover, there was a significant improvement of the endothelial-dependent vasomotion. Psychological stability as evaluated by the HADS questionnaire (depression and anxiety) also was improved. The rate of device dysfunction during CR may range up to 10% (e.g., over-sensing, myopotentials, etc.). Moreover, there is no evidence that exercise-based CR may reduce ICD-shock rates or reduce mortality of this population.

#### 3.7.3. Limitations

The clinical and prognostic effect of CR in ICD/CRT patients strongly depends on the underlying heart disease and the pathophysiological origins of arrhythmias. Restraints with respect to the individual aggregate, programming and implantation pocket need to be considered.

### 3.8. Patients with Ventricular Assist Device (VAD)

The following recommendations are based on the information delivered by newly performed semi-structured literature searches (classification: S2k, see Methods).

#### 3.8.1. Recommendations

- VAD implantation is recommended to be followed by cardiac rehabilitation conducted in specialized institutions (↑↑, 100%).
- These specialized CR-centers are recommended to guarantee the following preconditions (↑↑, 100%):
  - Profound knowledge with respect to the various VAD systems being used;
  - Close cooperation with the correspondent heart center;
  - Emergency equipment and regulations adapted to the special requirements of VAD-patients.
- CR of VAD-patients is recommended to include the following measures with respect to therapy, patient’s support and supervision (↑↑, 100%):
  - Individually adapted endurance training plus dynamic strength training of light to moderate intensity as recommended for patients with chronic heart failure;
  - Echocardiographic examinations of myocardial function need to include VAD-specific evaluations for rapid detection of VAD-dysfunction;
  - Daily monitoring for rapid detection and treatment of infections;
  - Regular training courses addressing patients and therapeutic team in order to improve knowledge and competence especially with respect to VAD-dysfunction and emergency situations;
  - Training of patients and their relatives in changing driveline dressings;
  - Training of eligible VAD-patients with respect to self-monitoring of anticoagulation;
  - Psychological support;
  - Vocational support and after-care in cooperation with the heart center responsible for the VAD implantation.
  - Guideline adjusted treatment of cardiac arrhythmias and supervision of additional devices like pacemakers, ICD and CRT.

#### 3.8.2. Scientific Evidence

In light of the complexity of VAD-systems and the severity of the underlying disease, a specialized care after VAD implantation is undisputable, and—according to existing guidelines—exercise-based CR is regarded to be safe [91,92,93,94]. CR including aerobic exercise training and dynamic stress training was associated with an increase in VO_2max_, 6-min walking test distance, and quality of life [95,96,97,98,99]. All studies reported cardiac rehabilitation as being safe, also being confirmed by the patient’s experience [100,101,102,103,104,105].

#### 3.8.3. Limitations

There are no studies reporting on the effect of CR on morbidity, mortality, psychosocial aspects and occupational reintegration.

### 3.9. Patients after Heart Transplantation (HTX)

The following recommendations are based on the information delivered by newly performed semi-structured literature searches (classification: S2k, see Methods).

#### 3.9.1. Recommendations

- During the first weeks after HTX cardiac rehabilitation is recommended (↑↑; 100%) to preferably be conducted as inpatient CR in close cooperation with the transplant center.
- Eligible CR centers are recommended (↑↑; 100%) to provide a special knowledge and clinical experience with respect to
  - Immunosuppressive medication and its potential drug–drug interactions;
  - Early recognition of transplant rejection;
  - Early recognition of infection.
- CR content is recommended to be adapted to the special needs of HTX-patients as outlined in the following (↑↑, 100%):
  - Exercise intensity needs to be adapted to the elevated resting heart rate after denervation resulting in a reduced heart rate reserve and consecutive limited cardiopulmonary performance;
  - The CR program has to include intense information and instructions on the effect of immunosuppressant drugs, their interaction with other drugs and the elevated risk of infection due to immunosuppressive medication;
  - Patients must be informed about occasionally discreet clinical signs of rejection;
  - Moreover, HTX-patients need to be counselled and motivated to reduce their individual cardiovascular risk by healthy nutrition, weight and blood pressure control as well as smoking cessation;
  - HTX-patients also need to have psychosocial support and counselling on vocational aspects if required;
- The inpatient CR-program is suggested to be followed by a long-term outpatient aftercare program (↑, 100%).

#### 3.9.2. Scientific Evidence

In small controlled studies “high-intensity interval training” (HIIT) was associated with a significant increase in VO_2max_ but without changes in LV-function [106].

Resistance exercise training in addition to “walking” also was associated with an increase in bone mineral density, whereas “walking” alone was not [107]. Moderate resistance exercise training also diminished the negative effects of immunosuppression on muscle mass and bone mineral density in HTX-patients [108]. Dynamic bicycle training significantly improved exercise capacity and quality of life, even when started 5 years after HTX [109]. A beneficial effect of exercise training on VO_2max_ and muscle strength was shown in a small meta-analysis including six studies, being confirmed by a recent Cochrane analyses (nine studies performed 1999–2015, *n* = 284 patients in total) [110,111]. Finally, on the basis of a controlled registry including *n* = 595 HTX-patients CR-participation was associated with a significantly reduced rehospitalization rate of 29% within one year [112].

#### 3.9.3. Limitations

There are no data regarding the effect of physical exercise training on the risk of graft vasculopathy. There also are no data describing prognostic long-term effects of multicomponent CR in HTX-patients. Finally, there are no data regarding the effect of CR on social and vocational reintegration of HTX-patients.

The present guidelines did not evaluate patients with systemic diseases leading to myocardial failure including potential consequences with regard to HTX. This field needs to be worked out by the next update of the guideline [113,114].

### 3.10. Patients after Surgical or Interventional Repair of the Aorta

The following recommendations are based on the information delivered by newly performed semi-structured literature searches (classification: S2k, see Methods).

#### 3.10.1. Recommendations

- Patients after surgical or interventional repair of the aorta are recommended to participate in a cardiovascular rehabilitation program (↑↑; 100%).
- During CR after surgical and interventional repair of the aorta the following conditions are recommended (↑↑; 100%):
  - All patients after surgical or interventional repair of the aorta need to have a risk evaluation at CR start as a basis to individually adapt the exercise program;
  - For medical blood pressure control all patients after repair of the aorta are to be treated with beta receptor blockers as a baseline medication;
  - Following risk evaluation and provided that blood pressure is sufficiently under control a supervised and a controlled stress-test is recommended at the start and end of CR.
  - During CR, blood pressure needs to be monitored closely including ambulatory blood pressure monitoring. During any stress test, the systolic blood pressure must not exceed 160 mmHg;
  - For physical exercise, ergometer training should be preferred as this allows repetitive blood pressure measurements;
  - Particular attention must be guaranteed with respect to signs and symptoms suspicious for typical complications like mal-perfusion (e.g., claudication, intestinal angina, repetitive back pain, hoarseness or increasing dysphagia).
  - Competitive sports, contact sport, sprinting, isometric strengths sports, and any physical effort associated with exhaling on exertion are to be strictly avoided!

#### 3.10.2. Scientific Evidence

In small prospective and retrospective studies evaluating patients after repair of aortic dissection, an individually adapted exercise training in various forms was shown to be safe and to be associated with a significant increase in exercise capacity. During exercise systolic blood pressures reached levels up to 150–170 mmHg or even more without complications but caution is still warranted and exercise levels remain to be individually adapted. Lowering blood pressure with beta-blockers or calcium antagonists was associated with a reduction in repeat surgery and improved survival [115,116,117,118,119].

#### 3.10.3. Limitations

There are no controlled studies evaluating the effect of cardiovascular rehabilitation on morbidity and mortality of patients after aortic dissection repair. Vocational reintegration may be supported by CR-participation, but data are scarce [115].

### 3.11. Patients with Peripheral Artery Disease (PAD)

The following recommendations are based on the information delivered by newly performed semi-structured literature searches (classification: S2k, see Methods).

#### 3.11.1. Recommendations

- Patients with peripheral artery disease (PAD) are recommended to participate in cardiovascular rehabilitation under the following conditions (↑↑; 100%):
  - PAD Stage IIa and IIb;
  - PAD Stage IIa following revascularization (surgical or interventional);
  - PAD Stage IIb following revascularization and in cases with delayed wound healing.
- Patients with peripheral artery disease (PAD) stage III of IV are not recommended to participate in cardiovascular rehabilitation (↓↓; 100%).
- In patients with PAD as a primary disease, CR is suggested to be delivered in specialized rehabilitation centers including the following standard equipment(↑; 100%):
  - Guideline-based PAD treatment concept;
  - Supervision by a medical specialist in angiology;
  - Exercise therapists being specialized in angiology;
  - Nursing staff skilled in wound management;
  - Physiotherapists skilled in manual measures and able to alleviate physical symptoms impairing the success of exercise training.
- Starting CR, the following primary diagnostics are recommended to be performed in all PAD patients (↑↑; 90%):
  - Assessment of peripheral pulses
  - Standardized walking distance test at the start and end of rehabilitation
  - Ankle-brachial index (ABI)
  - Time of re-capillarization;
  - color-coded duplex sonography
  - assessment of health-related quality of life
  - assessment of cardiovascular risk factors
  - assessment of psychosocial problems
- Treatment of PAD patients during CR is recommended to apply all measures needed for effective secondary prevention (↑↑; 100%), including:
  - individually adapted, intermittent walking exercise
  - exercise training to improve cardiopulmonary exercise capacity
  - modular and structured training courses for information, education and to support patients in dealing with their disease
  - smoking cessation courses
  - psychosocial support, if indicated
- During CR all PAD patients are suggested to participate in special vascular exercise training programs being separated from CAD-patients (↑; 100%).
- With respect to the social and vocational support of PAD-patients, it is recommended to consider potential limitations in walking capability (↑↑; 100%).
- Following CR all PAD-patients are recommended to follow the medical care by an angiologist and to participate in an ambulatory vascular sports group (↑↑; 100%).

#### 3.11.2. Scientific Evidence

Intermittent walking exercise is well documented to significantly improve the pain free walking distance as well as the health-related quality of life in PAD patients. Intermittent walking exercise in combination with a strict adherence to guideline recommended medication is associated with a reduction in cardiovascular mortality and rate of amputations in these patients. Moreover, psychosocial aspects need to be considered to increase the patient’s long-term adherence [120,121,122,123,124].

#### 3.11.3. Limitations

The clinical and prognostic effect of the short-term high intensity rehabilitation program as offered in Germany has not been validated so far.

### 3.12. Patients after Pulmonary Embolism (PE) with or without Deep Vein Thrombosis (DVT)

The following recommendations are based on the information delivered by newly performed semi-structured literature searches (classification: S2k, see Methods).

#### 3.12.1. Recommendations

- Patients after PE with or without DVT are suggested to participate in CR (↑, 100%).
- In patients after PE, a structured and supervised exercise is recommended, if the following preconditions are guaranteed (↑↑, 100%):
  - Guideline adjusted anticoagulation;
  - DVT compression of superficial veins.
- Starting CR, the following diagnostics are recommended in all patients after PE (↑↑, 100%):
  - Assessment of pulmonary artery blood pressure by transthoracic echocardiography to detect ongoing pulmonary hypertension and to follow the course of the disease;
  - Assessment of cardiopulmonary performance preferably by spiroergometry or alternatively by ergometry combined with determination of oxygen saturation;
  - 6-min walking test (including determination of oxygen saturation) in patients with low exercise capacity;
  - Evaluation of psychosocial as well as vocational issues.
- Therapeutic and educational measures during CR of patients after PE are recommended to include (↑↑; 100%):
  - Structured and supervised exercise training including individually adapted bicycle training, dynamic resistance training, aqua aerobics and gymnastics;
  - In patients with low exercise capacity, hand crank ergometer, gymnastics, and walking exercise may alternatively be used;
  - Psychosocial interventions, if necessary;
  - General educational programs focusing on risks, origins and pathophysiology of thrombosis, and in addition communicating how to prevent recurrent thrombosis including healthy lifestyle and anticoagulation.
  - Detailed information needs to be delivered with respect to (a) avoiding gravidity during anticoagulation in general, (b) using “low molecular heparins” instead of vitamin K antagonists or NOAK in case there is a wish to have children, (c) the elevated risk with respect to recurrent thrombosis by using oral contraceptives or postmenopausal estrogens
  - In patients suffering from neoplastic disorder anticoagulation shall be delivered according to the specific guidelines

#### 3.12.2. Scientific Evidence

Data of a retrospective study including *n* = 422 patients after pulmonary embolism and being under anticoagulation confirm the feasibility of exercise training and a low rate of bleeding complications. Another prospective study including *n* = 70 patients after PE showed comparable results. Moreover, the rate of fatal and non-fatal events including recurrent PE during CR and within a follow-up of twelve months was low. A small prospective and controlled study (intervention *n* = 9, control *n* = 10) showed a significant increase in VO_2max_ in the CR-group [125,126,127,128,129,130].

#### 3.12.3. Limitations

To date there are no randomized studies investigating prognostic effects of CR following pulmonary embolism. Moreover, the effect of quality of life and social parameters have not been investigated.

### 3.13. Patients with Pulmonary Hypertension (PH) of Various Origins

The following recommendations are based on the information delivered by newly performed semi-structured literature searches (classification: S2k, see Methods).

#### 3.13.1. Recommendations

- Patients with severe pulmonary hypertension and under optimized, guideline-adjusted medical treatment are suggested to participate in a structured and closely supervised exercise training (↑, 100%).
- Exercise training in patients with severe PH is suggested to preferably take place in a CR-center experienced in treating PH-patients in close cooperation with a PH expert center (↑, 100%).
- Starting CR, the health-related quality of life, individual risk behaviors and potential psychosocial problems are recommended to be evaluated and therapeutically addressed (↑↑, 100%).
- Exercise training is recommended to take place in small groups, being closely supervised and to be started in low doses individually adjusted in a daily manner
  (↑↑, 100%).
- Accordingly, all target parameters are suggested to be systematically checked during CR. In this way increased risks with respect to syncope, respiratory infects, hypoxemia, and arrhythmias are to be considered (↑, 100%).

#### 3.13.2. Scientific Evidence

Randomized controlled studies and controlled cohort studies providing a close in-hospital monitoring consistently showed a significant increase in exercise capacity as a result of individually adapted exercise training, a reduction in heart failure symptoms, and an increase in health-related quality of life [131,132,133,134,135,136,137,138,139,140]. Moreover, a reduction in pulmonary vascular resistance and significant increase in cardiac output has been shown [134]. In a recent prospective, randomized, controlled, multicenter study including *n* = 129 PH-patients out of 11 centers across 10 European countries (“pulmonary arterial” and “chronic thromboembolic pulmonary” hypertension), feasibility and safety of individualized exercise training in addition to medical therapy was confirmed. Exercise training was associated with a significant increase in 6-min walking distance and improvements in quality of life, WHO functional class and peak oxygen consumption. On the basis of these data, individualized exercise training in PH-patients can be regarded as an integral part of rehabilitation programs in these patients, provided sufficient medical supervision and expertise is guaranteed. [140].

#### 3.13.3. Limitations

To date there are no reliable data on the long-term prognostic effect of exercise-based CR in patients with pulmonary hypertension.

### 3.14. Patients after Myocarditis

The following recommendations are based on the information delivered by newly performed semi-structured literature searches (classification: S2k, see Methods).

#### 3.14.1. Recommendations

- In patients with “acute myocarditis” participation in rehabilitation programs is not recommended (↓↓, 100%). Intensive medical care and physical rest are indicated.
- In all other states, e.g., “subacute myocarditis”, “healed myocarditis” or “myocarditis in history”, participation in CR-programs is suggested (↑, 100%).
  In patients with “subacute myocarditis”, defined as
  - clinically stable patients;
  - laboratory markers of inflammation and myocardial injury/stress being in the normal range or continuously declining to almost normal values;
  - ECG without or with clearly declining pathological signs;
  - Echocardiogram presenting without or with declining pericardial effusion, and with normal or improving LV-function;
  - Holter-ECG presenting without clinically relevant arrhythmias.
- exercise training is suggested to start at “extremely low” levels (BORG scale 6-8/20)
  (↑, 100%), but a symptom-limited exercise test is not suggested to be performed at this stage (↓, 100%).
  In patients with “healed myocarditis”, defined as
  - clinically stable patients;
  - laboratory markers of inflammation and myocardial injury/stress in the normal range;
  - ECG normal or with stable residual changes like “bundle branch block”;
  - Echocardiogram presenting without or only minimal pericardial effusion and normal or only slightly reduced LV-function;
  - Holter-ECG presenting without clinically relevant arrhythmias.
- A symptom-limited exercise test is suggested before starting a regular structured and supervised exercise training of “low” intensity (BORG scale 10-12/20) (↑, 100%).
- A slowly intensifying exercise training may be considered (↔, 100%), but physical exertion should be avoided.
- CR in patients with “healed myocarditis” is recommended to include regular visits and controls of ECG, echocardiogram, and laboratory parameters to early detect potential adverse cardiac effects (↑↑, 100%).
- Competitive sports or other high grade physical activities are recommended not to start before 3–6 months after complete recovery (↑↑, 100%).
- Patients with “healed myocarditis” are recommended to be supported in their social and vocational reintegration (↑↑, 100%).
- In the case of prolonged cardiovascular symptoms and ongoing physical limitations despite inconspicuous cardiovascular diagnostics, it is recommended to consider psychological comorbidity (↑↑, 100%).

#### 3.14.2. Scientific Evidence and Limitations 

To date there are no controlled trials investigating the effect of CR in patients after acute myocarditis on clinical prognosis, health-related quality of life or psychosocial outcomes [141,142,143,144]. The recommendation of patients with “subacute” or “healed myocarditis” to participate in CR is primarily based on the need of a prolonged and close supervision and treatment with respect to

- remaining risks of high-grade arrhythmias especially after starting physical exercise;
- persistent myocardial failure;
- needs for psychosocial support.

### 3.15. Adults with Congenital Heart Disease (ACHD)

The following recommendations are based on the information delivered by actualized semi-structured literature searches (classification: S2k, see Section 2).

#### 3.15.1. Recommendations

- Adults with congenital heart disease (ACHD) are suggested to participate in cardiac rehabilitation programs (CR), if they had cardiac surgery, interventional treatment or if they suffer from complications of the underlying CHD (↑, 100%).
- CR of ACHD—especially in case of a complex disease—is suggested to be performed in rehabilitation centers familiar with the care of these patients and integrated in a medical network specialized in treating and supervising ACHD (“AHCD expert center”) (↑, 100%).
- For this reason, rehabilitations centers treating ACHD also are suggested to provide the presence and supervision of an adult/pediatric cardiologist with ACHD certification (↑ 100%).
- In detail, rehabilitation centers treating and supervising ACHD are recommended to meet the following requirements (↑↑100%):
  - Providing a multidisciplinary supervision and treatment of all cardiac and non-cardiac problems of ACHD;
  - Individual grading of the actual cardiovascular limitations and risks as the basis for proper allocation into the exercise programs;
  - Delivery of individualized and closely supervised exercise training taking into account the current cardiac status, potential CHD-related limitations in cardiac function including heart failure, pulmonary hypertension, arrhythmias, infective endocarditis, and pathologies of the aorta and others;
  - Health-related quality of life, individual risk behaviors and psychosocial problems are recommended to be systematically assessed at the beginning of CR and also need to be addressed by the individualized rehabilitation program.

#### 3.15.2. Scientific Evidence

Recommendations are based on a limited number of small, uncontrolled, and retrospective clinical trials showing individualized, structured and supervised exercise interventions for ACHD to be safe and enabling patients to significantly increase their physical performance. CR also has been shown to increase quality of life [145,146,147,148,149].

#### 3.15.3. Limitations

The population of ACHD is heterogeneous requiring a highly individual approach and thereby hampering large-scale controlled studies.

## 4. Summary and Critical Conclusions

The guidelines on cardiac rehabilitation (CR) in German speaking countries (LLKardReha-DACH) aim to summarize the actual scientific evidence with respect to CR-indications and CR-delivery and thereby contribute to improve patients’ clinical prognosis and to support their social and vocational reintegration. The participating countries are Germany, Austria and Switzerland. The extended original version of these guidelines was published online in 2020 by the “working group of the scientific medical societies” in Germany (www.awmf.org, 17 January 2021).

The English version of the guideline presented here concentrates on the evidence-based and consented recommendations, whereby part 1 presents and shortly discusses a broad range of different clinical indications for potential CR-referral.

Covering all potential indications for CR-referral as outlined in this publication there is consensus that CR-delivery in clinical practice needs to consider a large variety of clinical aspects and to guarantee the special expertise for a successful treatment, supervision and guiding of the patients.

There also is consensus that CR needs to deliver an individually adjusted minimum of “treatment intensity” including a strict reduction in cardiovascular risk factors, an individually adjusted minimal volume of physical exercise and last not least an individualized psychosocial support.

All these aspects strongly support the need for cardiac rehabilitation being multidisciplinary and medically supervised.

Based on these preconditions, there is strong evidence that CR-participation is associated with a significantly reduced mortality in patients after ACS and after CABG, whereas in patients with severe chronic systolic heart failure, the beneficial effect of CR-participation may be limited to a significantly increase in exercise capacity and improvement of health-related quality of life. In patients with chronic coronary syndrome and in individuals with high cardiovascular risk, a large variety of prevention programs show beneficial clinical effects, but minimal requirements to improve clinical prognosis are less well defined. This also is true for other cardiovascular diseases discussed in these guidelines.

However, apart from the anticipated improvement of clinical prognosis, the ability to participate in social life without severe restrictions also must be regarded as an individually important endpoint. This especially is true for a variety of indications discussed in these guidelines (e.g., patients with PAD, after surgery of the aorta, patients with transplanted heart or with VAD). Unfortunately, the effects of CR-participation on the endpoints “health-related quality of life” and “social and vocational reintegration” are not stringently investigated through all cardiovascular diseases and potential indications for CR-referral. This also is true for potential socioeconomic effects of CR-participation.

Against this background of remaining deficiencies in evidence a more focused scientific approach in rehabilitation science is warranted, thereby strictly reflecting minimal international standards in methodology, definition and documentation of populations, controls, interventions and outcomes.

## Figures and Tables

**Table 1:** Cardiac Rehabilitation Guidelines of German Speaking Countries in Europe (LLKardReha-DACH): Guidelines generation—major organizational steps.

**Step 1**	**Determination of Contents**: “Lead Management” and “Steering Committee”
**Step 2**	**Selection of leading authors with topic-related expertise**: “Lead management”
**Step 3**	**Internal control and reviewing of topic-related chapters**: “Lead management”
**Step 4**	**External control and reviewing of topic-related chapters,** **grading and final approvement of evidence-based recommendations**: “Steering committee”
	**External supervision:** AWMF; “Association of the Scientific Medical Societies in Germany”

**Table 2:** LLKardReha-DACH: evidence generation—classification of evidence.

Classification of Scientific Evidence According AWMF Rules	Evaluation of Scientific Evidence
**S3**	Data acquisition and evaluation: Systematic reviews and meta-analyses were newly performed on the basis of the PRISMA and the MOOSE statements [24,25]. The evaluation of included studies followed the Cochrane risk of bias table (http://tech.cochrane.org/revman/download, currently valid version, accessed on 24 January 2021) for RCTs and the Newcastle–Ottawa Scale (NOS) for controlled cohort studies (CCS) [26]. In addition, a new methodological approach for the evaluation of CCS was developed, applied and published [17]. Reviewing and publication of meta-analyses: Meta-analyses were published in peer-reviewed journals before their results were discussed and graded within the guideline [17,27,28,29]. The guideline text was internally reviewed by the “lead management” followed by external assessment and final judgement by the “steering committee”. Systematic grading of scientific evidence: The scientific evidence acquired by meta-analyses was graded following the Scottish Intercollegiate Guidelines Network (SIGN) grading system (see Supplemental Material) [30]. Consensus-based grading of recommendations: Clinical recommendations were determined in face-to-face sessions of the steering committee according to the definitions outlined in Table 3.
**S2k**	Data acquisition and evaluation: Scientific evidence was generated on the basis of the most actual and topic-related scientific guidelines, and in addition by a semi-structured evaluation of the scientific literature using PubMed and the Cochrane Library. Review process: All S2k chapters were internally pre-reviewed by the “lead committee” followed by the external and final judgement of the “steering committee” as supervised by the AWMF. Grading of evidence: As content and recommendations of S2k chapters were not based on newly performed meta-analyses, there was no formal grading of the underlying scientific evidence.
**Narrative evidence** **reporting, NER**	Data acquisition and evaluation: Scientific evidence was generated on the basis of the most actual and topic-related scientific guidelines. Review process: There was no formal and predefined review process, but all chapters were reviewed by expert members of the “lead management” and “steering committee”. Grading of evidence: There was no newly performed formal grading of the underlying scientific evidence. Scientific evidence as published in current guidelines were cited, reported and discussed.

**Table 3:** LLKardReha-DACH: grades of recommendation.

**Strong recommendation**	**“is recommended…”** **↑↑** **“is not recommended…”** **↓↓**
**Medium recommendation**	**“is suggested…”** **↑** **“is not suggested….”** **↓**
**neutral**	**“may be considered”** **↔**

## Data Availability

Not applicable.

## Supplementary Materials

The following information is available online at https://www.mdpi.com/article/10.3390/jcm10102192/s1, Data S1: Content of LL-KardReha-DACH in its original form in German language, Data S2: Classification of scientific evidence according to SIGN.

## Cardiac Rehabilitation Guidelines “Lead Management”:

- Schwaab, Bernhard
- Rauch, Bernhard
- Bjarnason-Wehrens, Birna
- Albus, Christian
- Meng, Karin
- Schmid, Jean-Paul
- Benzer, Werner
- Jensen, Katrin ^†^

## Officially Nominated Delegates of Participating Scientific Societies and Health Care Institutions (“Steering Committee”):

- Albus Christian, “Deutsches Kollegium für Psychosomatische Medizin“ (DKPM)
- Benzer Werner, “Österreichische Kardiologische Gesellschaft“ (ÖKG)
- Falk Johannes, “Deutsche Rentenversicherung Bund“ (DRV)
- Gielen Stephan, “Deutsche Gesellschaft für Kardiologie“ (DGK), Klinikum Lippe, Detmold, Germany
- Grande Gesine, “Deutsche Gesellschaft für Rehabilitationswissenschaften“ (DGRW), Brandenburgische Technische Universität Cottbus-Senftenberg (President), Germany
- Gysan Detlef, “Bundesverband der Niedergelassenen Kardiologen“ (BNK), University Witten-Herdecke, Germany
- Joisten Christine, “Deutsche Gesellschaft für Sport—und Präventivmedizin“ (DGSP), Germany
- Schmidt, Jean-Paul, “Swiss Working Group for Cardiovascular Prevention, Rehabilitation and Sports Cardiology”, SCPRS

## External Supervision of Evidence Generation and Grading:

“Arbeitsgemeinschaft der wissenschaftlichen medizinischen Fachgesellschaften” (“working group of the scientific medical societies” in Germany, AWMF; www.awmf.org accessed on 17 January 2021), represented by

- Kopp Ina B, AWMF-Institut für Medizinisches Wissensmanagement, Philipps-Universität, D-35043 Marburg, Germany
- Nothacker Monika, AWMF-Institut für Medizinisches Wissensmanagement, Philipps-Universität, D-35043 Marburg, Germany
- Blödt, Susanne, AWMF-Institut für Medizinisches Wissensmanagement, Geschäftsstelle Berlin, D-10559 Berlin, Germany

## Statistics and Biometry:

- Jensen Katrin (group leader) ^†^
- Metz Matthes
- Saure Daniel

## Management of Literature:

- Grilli Maurizio, M.L.I.S
- Metzendorf, Maria-Inti, Dipl. information specialist

## Additional Members of the LL-KardReha-DACH Topic Related Expert Panel Specifically Engaged in LLKardReha-DACH-Part 2:

- Bönner, G, Medizinische Fakultät Albert-Ludwigs-Universität zu Freiburg; D-79104 Freiburg, Germany
- Brzoska, P, Universität Witten/Herdecke, D-58448 Witten, Germany
- Buhr-Schinner, H, Ostseeklinik Schönberg-Holm, D-24217 Ostseebad Schönberg/Holstein, Germany
- Charrier, A, D-01328 Dresden, Germany
- Cordes, C, Gollwitzer-Meier-Klinik, D-32545 Bad Oeynhausen
- Exner, A-K, Klinikum Lippe GmbH, D-32756 Detmold, Germany
- Fromm, B, REHA-Klinik Sigmund Weil GmbH, D-76669 Bad Schönborn, Germany
- Gohlke H Neue Kirchstr.22, D-79282 Ballrechten-Dottingen, Germany
- Gysan, D, Department für Humanmedizin, Private Universität Witten/Herdecke GmbH, D58455 Witten, Germany
- Härtel, Ursula, LMU München, Institut für medizinische Psychologie, D-80336 München, Germany
- Hahmann, H, Ringweg 13, D-88316 Isny, Germany
- Herrmann-Lingen, C, Klinik für Psychosomatische Medizin und Psychotherapie, D-37075 Göttingen, Germany
- Karger, G, Rehaklinik Heidelberg-Königstuhl, D-69117 Heidelberg, Germany
- Karoff M, D-58332 Schwelm, Germany
- Kiwus, U, D-14169 Berlin, Germany
- Knoglinger, E, Kirchberg-Klinik, D-37431 Bad Lauterberg, Germany
- Krusch, C, Agentur für Arbeit Ludwigshafen, D-67059 Ludwigshafen, Germany
- Langheim, E, Reha-Zentrum Seehof der Deutschen Rentenversicherung Bund, D-14513 Teltow bei Berlin, Germany
- Mann, J, KfH Zentrum, D-80804 München-Schwabing, Germany
- Max, R, Zentrum für Rheumatologie, D-69115 Heidelberg, Germany
- Predel H-G, Institute for Cardiology and Sports Medicine, Dep. Preventive and Rehabilitative Sport- and Exercise Medicine, German Sport University Cologne, D-50933 Köln, Germany
- Preßler A, Privatpraxis für Kardiologie, Sportmedizin, Prävention, Rehabilitation, D-81675 München, Germany
- Razum, Oliver, Fakultät für Gesundheitswissenschaften, Universität Bielefeld, D-33615 Bielefeld, Germany
- Schütt M, Diabetologische Schwerpunktpraxis, D-23552 Lübeck, Germany
- Schultz, K, Klinik Bad Reichenhall, D-83435 Bad Reichenhall, Germany
- Skoda, Eva-Maria, Clinic for Psychosomatic Medicine and Psychotherapy, LVR University Hospital, University of Duisburg-Essen, D-45147 Essen, Germany,
- Steube, D, D-10704 Berlin, Germany
- Streibelt, Marco, Dr., German Federal Pension Insurance, Dep. for Rehabilitation Research, D-10704 Berlin, Germany
- Stüttgen, Martin, D-67157 Wachenheim, Germany
- Stüttgen, Michaela, ZAR Ludwigshafen Klinikum, D-67063 Ludwigshafen/Rhein, Germany
- Teufel, M, Clinic for Psychosomatic Medicine and Psychotherapy, LVR University Hospital, University of Duisburg-Essen, D-45147 Essen, Germany, D-45147 Essen, Germany
- Tschanz, H, Berner REHA Zentrum, CH-3625 Heiligenschwendi, Switzerland
- Vogel, H, Universität Würzburg Abteilung für Medizinische Psychologie und Psychotherapie, Medizinische Soziologie und Rehabilitationswissenschaften, D-97070 Würzburg, Germany
- von Schacky C, Omegametrix GmbH, D-82152 Martinsried, Germany

## Abbreviations

ACS	acute coronary syndrome
ATP	antitachycardia pacing
AWMF	Association of the Scientific Medical Societies in Germany
BORG/RPE	Borg Rating of Perceived Exertion Scale
CABG	coronary artery bypass grafting
CAD	coronary artery disease
CCS	controlled cohort studies
CCS	chronic coronary syndrome
CHF	chronic heart failure
CR	cardiovascular rehabilitation
CROS	Cardiac Rehabilitation Outcome Study
CRT	cardiac resynchronization therapy
COPD	chronic obstructive lung disease
CV	cardiovascular
CVD	cardiovascular disease
ECG	electrocardiogram
EF	ejection fraction
ESC-SCORE	European Society of Cardiology, Systematic COronary Risk Evaluation SCORE
HADS	Hospital Anxiety and Depression Scale
HFrEF	heart failure with reduced left ventricular ejection fraction
HIIT	high intensity interval training
HTX	heart transplantation
ICD	implanted cardioverter defibrillator
LV-EF	left ventricular ejection fraction
MET	metabolic equivalent
NOAC	new oral anticoagulants
NSTEMI	non ST-elevation myocardial infarction
NYHA	New York Heart Association
PAD	peripheral artery disease
PCI	percutaneous coronary intervention
PE	pulmonary embolism
RCT	randomized controlled trial
STEMI	ST-elevation myocardial infarction
VAD	ventricular assist device
WCD	Wearable Cardioverter-Defibrillator
