# Supplementary material for: Cardiac Rehabilitation in German Speaking Countries of Europe—Evidence-Based Guidelines from Germany, Austria and Switzerland LLKardReha-DACH—Part 1
Source: J Clin Med. 2021 May 19;10(10):2192. doi: 10.3390/jcm10102192 (PMC8161282; doi:10.3390/jcm10102192)
Supplement: Supplementary file 1 [file jcm-10-02192-s001.zip › jcm-1137028-supplementary.pdf]

Supplemental Material

Data S1. Content of LL-KardReha-DACH in its original form in German language

Table S1: LL-KardReha-DACH: Content and levels of evidence generation	
1. General information	- Initiation of the guideline and responsibilities<br>- Development, methods and internal reviewing processes
2. Introduction	- Aims of cardiac rehabilitation<br>- Definition of cardiac rehabilitation<br>- Legal regulations and CR-structures in Germany, Austria and Switzerland
3. General objectives of CR	- Goals and conflicting goals (NER)<br>- Cardiovascular prevention (NER)<br>- Psychosocial goals and tasks (NER)<br>- Social and vocational reintegration of patients (NER)
4. Indications for CR initiation and participation	- **after acute coronary syndrome (S3)**<br>- **after coronary bypass surgery (S3)**<br>- with chronic coronary syndrome (S2k)<br>- with high CV-risk (S2k)<br>- **with chronic heart failure (S3)**<br>- after surgical or interventional valve repair (S2k)<br>- after ICD/CRT implantation (S2k)<br>- with ventricular assist device (S2k)<br>- after heart transplantation (S2k)<br>- after surgical/interventional repair of the aorta (S2k)<br>- with chronic peripheral arterial disease (S2k)<br>- after pulmonary embolism (S2k)<br>- with chronic pulmonary hypertension (S2k)<br>- after myocarditis (S2k)<br>- adults with congenital heart disease (S2k)
5. CR-specific contents and interventions	- Medical supervision and nursing (S2k)<br>- Physical exercise modalities as adapted to the individually underlying cardiovascular diseases (S2k)<br>- **Psychological interventions (S3)**<br>- Smoking cessation (S2k)<br>- Nutrition (S2k)<br>- Ergotherapy (S2k)<br>- Social interventions (S2k)<br>- Information, education and training for lifestyle changes (S2k)

6. CV risk diseases and co-morbidities	- Hypertension (NER)<br>- Hyperlipidemia (NER)<br>- Diabetes and metabolic syndrome (NER)<br>- Adipositas (NER)<br>- Chronic renal disease (NER)<br>- Chronic obstructive lung disease (NER)<br>- Orthopaedic disorders (NER)<br>- Rheumatic diseases (NER)<br>- Psychiatric and neurological diseases (NER)
7. Special groups of patients in CR	- Old and frail patients (S2k)<br>- Young patients (S2k)<br>- Gender specialities (S2k)<br>- Migrants (S2k)
8. Medical aftercare, prevention programs	- Phase 3 programs (S2k)<br>- Integrated ambulatory health care and disease management programs (S2k)
9. Special concepts	- Work related prevention programs (S2k)<br>- Work related rehabilitation programs (S2k)<br>- Tele-rehabilitation and home-based rehabilitation (S2k)
10.	- Evidence – based quality assurance in CR (S2k)
**S3** = highest level of scientific evidence based on newly performed structured reviews and meta-analyses; **S2k** = medium level of scientific evidence based on semi-structured literature searches and evaluation without performing meta-analyses; **NER** = Narrative evidence reporting based on the most actual topic-related international guidelines	

Data S2. Classification of scientific evidence according to SIGN

Ref.: Scottish Intercollegiate Guidelines Network, SIGN 50, "A guideline developer's handbook". https://www.sign.ac.uk/pdf/sign50.pdf

LEVELS OF EVIDENCE

1 ++ High quality meta-analyses, systematic reviews of RCTs or RCTs with a very low risk of bias

1 + Well conducted meta-analyses, systematic reviews or RCTs with a low risk of bias

1 - Meta-analyses, systematic reviews or RCTs with a high risk of bias

2 ++ High quality systematic reviews of case control or cohort studies. High quality case control or cohort studies with a very low risk of confounding or bias and a high probability that the relationship is causal

2 + Well conducted case control or cohort studies with a low risk of confounding or bias and a
moderate probability that the relationship is causal

2 - Case control or cohort studies with a high risk of confounding or bias and a significant risk
that the relationship is not causal

3 Non-analytic studies, e.g. case reports, case series

4 Expert opinion
